# Supplementary material for: Exact Combinatorial Density of States for the Critical 1D Ising Model
Source: Entropy (Basel). 2026 Jul 19;28(7):821. doi: 10.3390/e28070821 (PMC13409586; doi:10.3390/e28070821)
Supplement: Supplementary file 1 [file entropy-28-00821-s001.zip › entropy-4432430-supplementary.pdf]

Supplemental Material: "Exact Combinatorial Density of States for the Critical 1D Ising Model"

Bastian Castorene,[1,2] Francisco J. Peña,[3,*] Martin HvE Groves,[1,2] and Patricio Vargas[2]

[1]*Instituto de Física, Pontificia Universidad Católica de Valparaíso, Casilla 4950, 2373223 Valparaíso, Chile*
[2]*Departamento de Física, Universidad Técnica Federico Santa María, 2390123 Valparaíso, Chile*
[3]*Facultad de Ingeniería, Universidad San Sebastián, Lago Panguipulli 1390, Puerto Montt, Chile*

This supplemental material provides the complete numerical verification of the exact combinatorial degeneracy formulas derived in the main text for both the open-chain and closed-ring topologies of the one-dimensional antiferromagnetic Ising model at the exceptional point $B/J = 2$. Three independent methods—brute-force enumeration, the combinatorial formula based on Fibonacci convolutions, and the transfer-matrix formalism—are systematically compared across the full excitation spectrum. The completeness relation $\sum \Omega = 2^N$ is explicitly verified in each case.

PACS numbers:

I. OVERVIEW OF VERIFICATION METHODS

To establish the exactness of the derived degeneracy formulas, we employ three independent computational approaches:

Method I: Brute-force enumeration via the Ising Hamiltonian. All 2^N spin configurations $\{\sigma_1, \ldots, \sigma_N\}$ with $\sigma_i \in \{-1, +1\}$ are explicitly generated. For each configuration, the full Hamiltonian

$$\mathcal{H} = J \sum_{\langle i,j \rangle} \sigma_i \sigma_j + B \sum_{i=1}^{N} \sigma_i \tag{1}$$

is evaluated at $B/J = 2$, where the nearest-neighbor sum runs over open-chain or periodic-ring bonds. The ground-state energy $E_0 = \min(\mathcal{H})$ is identified, and each configuration is classified by its excitation quantum number:

$$m^{\text{chain}} = (E - E_0)/(2J), \tag{2}$$
$$k^{\text{ring}} = (E - E_0)/(4J). \tag{3}$$

The degeneracy at each level is obtained by counting. This procedure provides an exact ground truth that is completely independent of any combinatorial or algebraic construction, directly resolving the physical Hamiltonian without intermediate abstractions.

Method II: Combinatorial formula. The explicit Fibonacci-convolution formula derived in the main text:

$$\Omega_m^{\text{chain}}(N) = \sum_{\substack{b+2k=m \\ b \in \{0,1,2\}}} \gamma_b \, D(N, b, k), \tag{4}$$

$$\Omega_k^{\text{ring}}(N) = \sum_{c=1}^{k} \binom{k-1}{c-1} \frac{N}{c} \mathcal{C}_c(N - k - c), \tag{5}$$

with $D(N, b, k)$ built from the boundary-extended Fibonacci convolutions $\widetilde{\mathcal{C}}_p^{(b)}(V)$.

Method III: Transfer matrix. The algebraic generating function approach:

$$\Omega_m^{\text{chain}}(N) = [y^m] \, \mathbf{v}_L \cdot \mathbf{T}^{N-1} \cdot \mathbf{v}_R, \tag{6}$$
$$\Omega_k^{\text{ring}}(N) = [y^{2k}] \, \text{Tr}(\mathbf{T}^N), \tag{7}$$

where $\mathbf{T} = \left(\begin{smallmatrix} 1 & 1 \\ 1 & y^2 \end{smallmatrix}\right)$ and $\mathbf{v}_L = \mathbf{v}_R^T = (1, y)$.

II. SPECTRAL STRUCTURE AND PHYSICAL BOUNDS

A. Open chain

For the open chain of N sites, the excitation quantum number $m = b + 2k$ ranges from $m = 0$ (ground state, $b = 0$, $k = 0$) to $m = 2N$ (the fully polarized all-up configuration, $b = 2$, $k = N - 1$). The spectrum contains exactly $2N - 1$ non-zero energy levels, since the levels $m = 2N - 2$ and $m = 2N - 1$ are always empty: no valid spin configuration can produce these excitation numbers.

The completeness relation reads:

$$\sum_{m=0}^{2N} \Omega_m^{\text{chain}}(N) = 2^N. \tag{8}$$

B. Closed ring

For the periodic ring of N sites, the excitation quantum number k (number of adjacent up-up pairs under PBC) ranges from $k = 0$ to $k = N$, where $k = N$ corresponds to the unique all-up configuration. The completeness relation is:

$$\sum_{k=0}^{N} \Omega_k^{\text{ring}}(N) = 2^N. \tag{9}$$

*Electronic address: francisco.pena@uss.cl

III. FULL VERIFICATION TABLES

A. Open Chain, $N = 10$: Three-Method Comparison

Table I presents the complete excitation spectrum of the open chain with $N = 10$ sites. All $2^{10} = 1024$ configurations are accounted for. The three methods agree exactly at every excitation level.

TABLE I: Complete excitation spectrum of the open chain at $N = 10$. All three methods—brute-force (BF), combinatorial formula (Comb), and transfer matrix (TM)—produce identical results. The Diophantine classes (b, k) satisfying $b + 2k = m$ are listed for each level. The levels $m = 18$ and $m = 19$ are empty; $m = 20$ corresponds to the unique all-up state.

m	$\Delta E/J$	Classes (b, k)	BF	Comb	TM	Match
0	0	$(0, 0)$	55	55	55	✓
1	2	$(1, 0)$	68	68	68	✓
2	4	$(0, 1)$, $(2, 0)$	92	92	92	✓
3	6	$(1, 1)$	118	118	118	✓
4	8	$(0, 2)$, $(2, 1)$	106	106	106	✓
5	10	$(1, 2)$	124	124	124	✓
6	12	$(0, 3)$, $(2, 2)$	96	96	96	✓
7	14	$(1, 3)$	94	94	94	✓
8	16	$(0, 4)$, $(2, 3)$	73	73	73	✓
9	18	$(1, 4)$	62	62	62	✓
10	20	$(0, 5)$, $(2, 4)$	44	44	44	✓
11	22	$(1, 5)$	26	26	26	✓
12	24	$(0, 6)$, $(2, 5)$	29	29	29	✓
13	26	$(1, 6)$	16	16	16	✓
14	28	$(0, 7)$, $(2, 6)$	8	8	8	✓
15	30	$(1, 7)$	2	2	2	✓
16	32	$(0, 8)$, $(2, 7)$	8	8	8	✓
17	34	$(1, 8)$	2	2	2	✓
18	36	—	0	0	0	✓
19	38	—	0	0	0	✓
20	40	$(2, 9)$	1	1	1	✓
		$\sum_{m=0}^{20}$	1024	1024	1024	$= 2^{10}$

B. Closed Ring, $N = 10$: Three-Method Comparison

Table II presents the complete excitation spectrum of the closed ring with $N = 10$ sites. The ring spectrum is strictly quantized in units of $4J$ and exhibits no boundary-related fine structure.

C. Open Chain, $N = 20$: Three-Method Comparison

For $N = 20$, brute-force enumeration of all $2^{20} = 1{,}048{,}576$ configurations via the full Ising Hamiltonian is included as an independent verification. Table III compares all three methods across the full spectrum.

TABLE II: Complete excitation spectrum of the closed ring at $N = 10$. All three methods agree exactly. The ground state ($k = 0$) yields the Lucas number $L_{10} = 123$.

k	$\Delta E/J$	BF	Comb	TM	Match
0	0	123	123	123	✓
1	4	210	210	210	✓
2	8	230	230	230	✓
3	12	190	190	190	✓
4	16	135	135	135	✓
5	20	70	70	70	✓
6	24	45	45	45	✓
7	28	10	10	10	✓
8	32	10	10	10	✓
9	36	0	0	0	✓
10	40	1	1	1	✓
$\sum_{k=0}^{10}$		1024	1024	1024	$= 2^{10}$

TABLE III: Complete excitation spectrum of the open chain at $N = 20$. All three methods—brute-force (BF), combinatorial formula (Comb), and transfer matrix (TM)—produce identical results across all $2N + 1 = 41$ excitation levels. Selected levels shown; the full spectrum sums to $2^{20} = 1{,}048{,}576$.

m	$\Delta E/J$	BF	Comb	TM	Match
0	0	6765	6765	6765	✓
1	2	8362	8362	8362	✓
2	4	22868	22868	22868	✓
3	6	28812	28812	28812	✓
4	8	44990	44990	44990	✓
5	10	55600	55600	55600	✓
6	12	65750	65750	65750	✓
7	14	77830	77830	77830	✓
8	16	78256	78256	78256	✓
9	18	87234	87234	87234	✓
10	20	79484	79484	79484	✓
15	30	48798	48798	48798	✓
20	40	15836	15836	15836	✓
25	50	2134	2134	2134	✓
30	60	274	274	274	✓
35	70	2	2	2	✓
38	76	0	0	0	✓
39	78	0	0	0	✓
40	80	1	1	1	✓
$\sum_{m=0}^{40}$			1,048,576		$= 2^{20}$

D. Closed Ring, $N = 20$: Three-Method Comparison

Table IV presents the ring spectrum for $N = 20$, where all three methods—including the brute-force enumera-

tion of 2^{20} configurations via the full Hamiltonian—are compared.

TABLE IV: Complete excitation spectrum of the closed ring at $N = 20$. All three methods agree exactly. The ground state yields $L_{20} = 15{,}127$. The maximum degeneracy occurs at $k = 4$ ($\Omega_4 = 165{,}490$). Note the empty level at $k = 19$ and the spectral gap below the fully polarized state at $k = N$.

k	$\Delta E/J$	BF	Comb	TM	Match
0	0	15127	15127	15127	✓
1	4	51680	51680	51680	✓
2	8	100590	100590	100590	✓
3	12	143580	143580	143580	✓
4	16	165490	165490	165490	✓
5	20	161824	161824	161824	✓
6	24	138250	138250	138250	✓
7	28	105100	105100	105100	✓
8	32	72095	72095	72095	✓
9	36	44820	44820	44820	✓
10	40	25694	25694	25694	✓
11	44	13220	13220	13220	✓
12	48	6585	6585	6585	✓
13	52	2680	2680	2680	✓
14	56	1270	1270	1270	✓
15	60	340	340	340	✓
16	64	190	190	190	✓
17	68	20	20	20	✓
18	72	20	20	20	✓
19	76	0	0	0	✓
20	80	1	1	1	✓
$\sum_{k=0}^{20}$			1,048,576		$= 2^{20}$

IV. DISCUSSION OF RESULTS

A. Exactness of the combinatorial formulas

The tables presented above demonstrate that the combinatorial formulas Eqs. (4) and (5) reproduce the exact degeneracy at every excitation level, for both topologies and across all system sizes tested. The brute-force enumeration—which directly evaluates the full Ising Hamiltonian $\mathcal{H} = J\sum \sigma_i\sigma_j + B\sum \sigma_i$ for all 2^N spin configurations with $\sigma_i \in \{-1, +1\}$—constitutes a completely independent verification that involves no combinatorial abstractions whatsoever. The exact agreement at both $N = 10$ and $N = 20$ (all four tables) provides a direct proof of correctness for each (N, m) or (N, k) pair examined.

The transfer-matrix method provides a second, algebraically independent verification. Because the generating polynomial $Z_N(y)$ is computed through matrix exponentiation—a procedure that involves no Fibonacci sequences, convolutions, or Diophantine classifications—the agreement between Methods II and III confirms that the combinatorial decomposition into topological classes is not merely a formal identity but captures the exact partition of the Hilbert space.

B. Spectral structure of the open chain

A noteworthy structural feature is the existence of two empty levels at $m = 2N - 2$ and $m = 2N - 1$ in the open-chain spectrum. These gaps arise because the configuration closest to the fully polarized state ($m = 2N$, the all-up state with $b = 2$, $k = N - 1$) requires flipping at least three excitation units to produce a distinct configuration: one must either remove a boundary excitation (changing m by -1) and simultaneously break an adjacent pair, or remove two adjacent pairs ($m \to m-4$). No configuration exists at $m = 2N - 2$ or $m = 2N - 1$, leaving a spectral gap of width $\Delta m = 3$ below the fully polarized state.

The total number of distinct non-zero energy levels is therefore $2N - 1$, distributed as $m \in \{0, 1, \ldots, 2N - 3\} \cup \{2N\}$.

C. Chain versus ring comparison

The chain and ring spectra exhibit fundamentally different structures reflecting their distinct topologies:

The open chain spectrum is quantized in units of $2J$, yielding a finer energy resolution due to the availability of fractional boundary excitations ($\Delta E = 2J$ per boundary spin flip). The closed ring, lacking boundary degrees of freedom, is strictly quantized in units of $4J$, producing a coarser but more symmetric spectrum.

For $N = 10$, the chain distributes its $2^{10} = 1024$ configurations across 19 non-zero levels, while the ring distributes them across 10 non-zero levels. The peak degeneracy occurs at $m = 5$ ($\Omega_5 = 124$) for the chain and at $k = 2$ ($\Omega_2 = 230$) for the ring, reflecting the ring's tendency to concentrate states at lower excitation levels due to the absence of boundary-mediated excitations.

For $N = 20$, the peak shifts to $m = 9$ ($\Omega_9 = 87{,}234$) for the chain and $k = 4$ ($\Omega_4 = 165{,}490$) for the ring. The ring consistently achieves higher peak degeneracies because its 2^N configurations are compressed into fewer excitation levels.

D. Completeness and the Hilbert space partition

The completeness relation $\sum \Omega = 2^N$ has been verified exactly for both topologies at $N = 10$ and $N = 20$, using all three methods independently. This identity is not merely a consistency check but reflects a fundamental physical requirement: the combinatorial formulas must partition the entire 2^N-dimensional Hilbert space into disjoint energy eigenspaces. The agreement confirms that no configurations are missed or double-counted by

the Diophantine classification and cluster-decomposition procedure. The inclusion of the brute-force Hamiltonian enumeration at $N = 20$ ($2^{20} = 1{,}048{,}576$ configurations) further strengthens this verification by providing a ground truth that is entirely independent of both the combinatorial and transfer-matrix formalisms.

E. Computational reproducibility

The Wolfram Mathematica notebook `NumericalValidation.m`, provided alongside this supplemental material, implements all three verification methods and reproduces every entry in Tables I–IV. The brute-force module directly evaluates the Ising Hamiltonian for all 2^N spin configurations with $\sigma_i \in \{-1, +1\}$, providing a ground truth that is completely independent of the analytical formulas. The notebook is structured in self-contained sections that can be executed independently:

1. Section 1: Brute-force enumeration via the full Ising Hamiltonian (chain and ring).
2. Section 2: Combinatorial formula for the open chain, Eq. (4).
3. Section 3: Combinatorial formula for the closed ring, Eq. (5).
4. Section 4: Transfer-matrix analytical diagonalization ($\mathbf{T} = \mathbf{P}\boldsymbol{\Lambda}\mathbf{P}^{-1}$).
5. Sections 5–6: Three-method verification tables for $N = 10$ and $N = 20$ (chain and ring).
6. Sections 7–10: Explicit formula verification, topological decomposition, spectra, and diagonalization check.
